# Activation of AMPK-PGC-1α pathway ameliorates peritoneal dialysis related peritoneal fibrosis in mice by enhancing mitochondrial biogenesis

**DOI:** 10.1080/0886022X.2022.2126789

**Published:** 2022-09-23

**Authors:** Jun Wu, Jushuang Li, Baohong Feng, Zhimin Bi, Geli Zhu, Yanxia Zhang, Xiangyou Li

**Affiliations:** Department of Nephrology, Tongren Hospital of Wuhan University (Wuhan Third Hospital), Wuhan University, Wuhan, P.R. China

**Keywords:** Peritoneal dialysis, peritoneal fibrosis, mitochondrial biogenesis, AMPK, PGC-1α

## Abstract

**Background:**

The pathogenesis of peritoneal dialysis (PD)-related peritoneal fibrosis (PF) is not clearly understood, and current treatment options are limited.

**Methods:**

In this study, the effect of PD-related PF on mitochondrial biogenesis was investigated, and the effect of activation of the adenosine monophosphate-activated protein kinase (AMPK)-PGC-1α (peroxisome proliferator-activated receptor γ coactivator-1α) pathway on PF was evaluated in mice.

**Results:**

In a mouse model of PD-related PF, AMPK-PGC-1α signaling (phospho-AMPK, PGC-1α, NRF-1, NRF-2 and TFAM expression) was downregulated, mitochondrial DNA (mtDNA) levels were reduced, and mitochondrial structure was damaged in the peritoneum. In addition, TdT-mediated dUTP nick-end labeling (TUNEL) staining showed typical apoptosis characteristics in peritoneal mesothelial cells (PMCs). Activation of the AMPK-PGC-1α pathway (PGC-1α overexpression or metformin, which is an agonist of AMPK) upregulated phospho-AMPK, PGC-1α, nuclear respiratory factors 1 (NRF-1) and 2 (NRF-2), and mitochondrial transcription factor A (TFAM) expression and mtDNA content, improved mitochondrial morphological manifestations, inhibited apoptosis of PMCs and alleviated PF.

**Conclusion:**

Our study may suggest that activation of the AMPK-PGC-1α pathway ameliorates PD-related PF by enhancing mitochondrial biogenesis.

## Introduction

Peritoneal dialysis (PD) has been an alternative therapy to classical hemodialysis procedures for end-stage renal disease (ESRD) patients. Currently, PD accounts for approximately 11% of all types of renal replacement therapy (RRT) around the world [1,2]. This modality of RRT relies on the structural and functional integrity of the peritoneum. Peritoneal fibrosis (PF) is a serious complication that results in ultrafiltration failure and decreased small solute clearance during PD practice. Signs of fibrosis are observed in 50–80% of ESRD patients in the early stage of PD [3,4].

Long-term exposure to conventional bioincompatible PD solutions results in histological changes in the peritoneal membrane, including loss of the peritoneal mesothelial cell (PMC) monolayer accompanied by excessive extracellular matrix (ECM) deposition and angiogenesis [5]. There are two cooperative parts of peritoneal fibrosis, including the fibrosis process itself and peritoneal inflammation caused by the nonphysiologic content of PD fluids and infections [3,6,7]. PMCs can transdifferentiate into fibroblastoid cells and produce ECM. The origin of peritoneal inflammation begins with various injuries to PMCs, which leads to macrophage recruitment. Macrophages participate in peritoneal inflammation with direct fibrotic consequences [8]. During the progression of peritoneal fibrosis, key fibrotic factors interact with their membrane receptors and activate downstream intracellular signal cascades, including transforming growth factor-β (TGF-β) and mitogen-activated protein kinase (MAPK), to trigger the transcription factors that activate cell matrix gene transcription [4]. However, the exact mechanism of PF is not completely understood, and it is necessary to identify new therapeutic targets to delay the progression of PF.

Mitochondria are multifunctional organelles and play a crucial role in maintaining cellular homeostasis. Mitochondrial dysfunction is associated with many pathological conditions [9,10]. Therefore, the maintenance of adequate and functional mitochondrial abundance is of vital importance. The mitochondrial dynamic balance is essential between mitochondrial fusion and fission, quality control systems and mitochondrial biogenesis [11]. Mitochondrial biogenesis is defined as a process by which cells increase their mitochondrial mass. Mitochondrial biogenesis is a complex process that involves the following steps: synthesis of inner and outer mitochondrial membranes, mitochondrial-encoded protein synthesis, nuclear-encoded mitochondrial protein synthesis and replication of mitochondrial DNA (mtDNA). The master regulator of mitochondrial biogenesis is composed of peroxisome proliferator-activated receptor γ coactivator-1α (PGC-1α), nuclear respiratory factors 1 (NRF-1) and 2 (NRF-2), and mitochondrial transcription factor A (TFAM) [12].

Recent studies have shown that mitochondrial dysfunction participates in the process of cardiac fibrosis, pulmonary fibrosis, renal fibrosis and liver fibrosis, and the study of mitochondria will likely yield potential therapeutic strategies for treating tissue organ fibrosis [13–16]. Interestingly, Inoue et al. [17] reported that mitochonic acid-5 ameliorated peritoneal fibrosis by suppressing macrophage infiltration and oxidative stress, which can restore mitochondrial function in mice. Li et al. [18] reported that Astragalus total saponins inhibited peritoneal fibrosis by increasing the mitochondrial membrane potential and restoring mitochondrial synthesis protein expression (PGC-1α, NRF-1 and TFAM).

In this study, the effect of PD-related PF on mitochondrial biogenesis was investigated, and the effect of activation of the adenosine monophosphate-activated protein kinase (AMPK)-PGC-1α pathway on PF was evaluated in mice. This study may provide a basis for a novel therapeutic strategy for ameliorating PF in long-term PD.

## Materials and methods

### Reagents

Metformin was obtained from Sigma–Aldrich (Merck KGaA, Darmstadt, Germany). The antibodies used in this study were from Cell Signaling Technology (Danvers, MA; anti-AMPK (#2532) and phospho-AMPK (Thr172, #2535), anti-NRF-1 (#69432) and NRF-2 (#20733) for immunoblotting); Abcam (Cambridge, UK; anti-PGC-1α (ab191838) and TFAM (ab272885) for immunoblotting; anti-NRF-1 (ab175932) and NRF-2 (ab31163) for immunohistochemistry); Santa Cruz Biotechnology (anti-Fibronectin (sc-8422), α-smooth muscle actin (α-SMA, sc-53142) and E-cadherin (sc-8426) for immunoblotting). A TGF-β_1_ enzyme-linked immunosorbent assay (ELISA) kit was purchased from Shanghai Enzyme-linked Biotechnology Co. Ltd. (Shanghai, China). The peritoneal dialysis fluid (4.25% Dianeal) was purchased from Baxter (Guangzhou, China).

### Animal models of PF

The animal model of PD-induced PF was established as described previously [19,20]. A total of 40 male C57BL/6 mice (12 weeks old, 28–32 g) were obtained from the Animal Research Center of Wuhan University. The mice were kept in standard cages and maintained under the following housing conditions: 12 h light-dark cycle, 22 ± 2 °C and 45–60% humidity. Food and water were freely available, and mice were allowed full mobility during the whole course of the study. A total of 40 mice were divided into 5 groups and were acclimatized for one week before experiments. Eight mice were intraperitoneally injected with 1.5 mL of 0.9% saline solution per day for 28 consecutive days (Control group). Eight mice were intraperitoneally injected with 1.5 mL of 4.25% Dianeal per day for 28 consecutive days (PF group). Eight mice were intraperitoneally injected with 1.5 mL of 4.25% Dianeal and AMPK agonist (metformin, 50 mg/kg/day) per day for 28 consecutive days (PF + Met group). Eight mice were intraperitoneally injected with 1.5 mL of 4.25% Dianeal per day for 28 consecutive days and were intraperitoneally injected with 500 μl suspensions containing 1 × 10^9^ plaque-forming units (PFU) of an adenovirus expressing active PGC-1α or empty adenoviral vector as the control on the 22nd day (PF + PGC-1α group, PF + vector group). The visceral and parietal peritoneal membrane and peritoneal dialysis effluent were collected. All experimental protocols involving mice received approval from the Animal Ethics Committee of Tongren Hospital affiliated with Wuhan University (approval number: SY2020-029).

### Adenovirus-mediated overexpression of PGC-1α

To overexpress PGC-1α in a mouse model of PF, an adenovirus vector expressing PGC-1α and control virus were constructed and packaged by Hanbio Biotechnology (Shanghai, China). The adenovirus was produced by cotransfecting human embryonic kidney (HEK)-293T cells with adenovirus transfer vector (pAdEasy, Hanbio Biotechnology) and packaging plasmids (pHBAd-BHG, Hanbio Biotechnology) using Lipofectamine 2000 (Thermo Fisher Scientific, Inc.). Briefly, HEK-293T cells were incubated at 37 °C in a humidified 5% CO_2_ atmosphere overnight. At the time of transfection, the confluency of the cells was ensured to be at least 70%. The medium was replaced by Dulbecco’s modified Eagle’s medium (Gibco, Thermo Fisher Scientific, Inc.) containing 10% fetal bovine serum (Gibco, Thermo Fisher Scientific, Inc.) 2 h before transfection. The transfer vectors (2 μg) and packaging plasmids (4 μg) were mixed with Lipofectamine 2000 (15 μL). The mixture was added to the culture dish for 6 h of incubation at 37°C in 5% CO_2_. After incubation for 1 day, the enhanced green fluorescent protein (EGFP) expression was visualized (green fluorescence) using a fluorescence microscope, which confirmed the successful transfection of PGC-1α into HEK-293T cells. Cell culture supernatants were harvested and cleared by centrifugation and filtration. All samples were fractioned and stored at −80 °C. The adenovirus was subsequently identified by sequencing and polymerase chain reaction (PCR) amplification. The primers for PGC-1α were CAATTGAAGAGCGCCGTGT (forward) and CCATCATCCCGCAGATTTAC (reverse). Finally, the adenovirus titer was measured using the 50% tissue culture infective dose (TCID50) assay. The pAdEasy-PGC-1α-EGFP virus (1.99 × 10^10^PFU/mL) and control virus (5.01 × 10^10^PFU/mL) were used.

### Immunoblot analyses

The visceral peritoneal membrane (mesentery tissue 50 mg) was collected. The protein concentrations of tissue lysates were measured using the Bradford method. A total of 20–25 μg of protein was subjected to sodium dodecyl sulfate (SDS)-polyacrylamide minigel and transferred to a nitrocellulose membrane. Nonspecific protein binding was blocked with 5% nonfat milk, followed by incubation with the primary antibodies overnight (AMPK 1:1000, phospho-AMPK 1:1000, PGC-1α 1:1000, NRF-1 1:1000, NRF-2 1:1000, TFAM 1:1000, fibronectin 1:1000, E-cadherin 1:1000, α-SMA 1:1000). After rinsing with 1x Tris buffered saline (TBS)-Tween 20, the membrane was incubated with the appropriate secondary antibody (1:2000). After washing, the protein was detected using an enhanced chemiluminescence system (Pierce, Thermo Fisher Scientific, Inc.).

### Real-time polymerase chain reaction

The visceral peritoneal membrane (mesentery tissue 50 mg) was collected. Total DNA was isolated from peritoneal tissue using the DNeasy Blood & Tissue kit (Qiagen) according to the manufacturer’s instructions. Total RNA was extracted from peritoneal tissue with TRIzol reagent according to the manufacturer’s instructions. Real-time polymerase chain reaction (RT–PCR) was performed using the ABI 7900HT sequence detection system (Applied Biosystems). The following primers were used. GGCCTGACTGGCATTGTATT (forward) and TGGCGTAGGTTTGGTCTAGG (reverse) for mitochondrial DNA-encoded cytochrome c oxidase subunit I (COX I), GCCGACTAAATCAAGCAACA (forward) and CAATGGGCATAAAGCTATGG (reverse) for COX II, TAGAGGGACAAGTGGCGTTC (forward) and CGCTGAGCCAGTCAGTGT (reverse) for nucleus-encoded 18S ribosomal DNA(18S rDNA). CAATTGAAGAGCGCCGTGT (forward) and CCATCATCCCGCAGATTTAC (reverse) for PGC-1α and GGGAAACTGTGGCGTGAT (forward) and GAGTGGGTGTCGCTGTTGA (reverse) for glyceraldehyde-3-phosphate dehydrogenase (GAPDH). Data were analyzed using the 2^−ΔΔ^*^Ct^* method. The relative mitochondrial copy number was evaluated by calculating the ratio of COX I or COX II to 18S rDNA.

### Immunohistochemistry

The parietal peritoneal membrane was fixed with 4% paraformaldehyde and embedded in paraffin. Tissue sections (3 μm in thickness) were used for immunohistochemical staining. The sections were deparaffinised in xylene and rehydrated with graded ethanol. After antigen retrieval, the sections were treated with 3% H_2_O_2_ for 10 min and blocked with 10% goat serum for 1 h. Then, the sections were incubated with the primary antibodies overnight. After rinsing with PBS, the sections were incubated with biotinylated secondary antibodies (Santa Cruz Biotechnology) for 1 h. Finally, the sections were washed and incubated with a diaminobenzidine (DAB) solution (DAB Peroxidase Substrate Kit, Vector). Tissue sections were examined and photographed under a light microscope.

### Morphological analyses of peritoneum

The parietal peritoneal membrane was fixed with 4% paraformaldehyde and embedded in paraffin. Parietal peritoneum sections of 3 μm thickness were stained with Masson’s Trichrome Stain Kit according to the manufacturer’s instructions (Beijing Solarbio Science & Technology Co., Ltd.). Sections were viewed and photographed under a light microscope, and the thickness of the parietal peritoneal membrane was measured with an Aperio ScanScope CS Slide Scanner system (Aperio Technologies, Vista, USA).

### TUNEL assay

Parietal peritoneal tissue sections (3 μm in thickness) were used for the TdT-mediated dUTP nick-end labeling (TUNEL) assay. The TUNEL assay was performed using the Cell Death Detection Kit (Roche, Penzberg, Germany) according to the manufacturer’s instructions. Digital images were obtained with a fluorescence microscope (Olympus, Japan). We selected 10 representative fields from each peritoneal tissue section and calculated the number of TUNEL-positive cells per 100 mm^2^.

### Transmission electron microscopy

Parietal peritoneal tissue sections were used for transmission electron microscopy (TEM) analyses. Briefly, peritoneal tissue sections were fixed with 2% glutaraldehyde and postfixed in 1% OsO4. Then, the sections were rehydrated with graded ethanol, followed by embedding and polymerization. A specimen of the parietal peritoneum was cut into ultrathin sections (60 nm in thickness). The sections were stained with uranyl acetate and lead citrate. Preparations were observed with TEM (HT7700, Hitachi, Japan).

### Enzyme-linked immunosorbent assay

The peritoneal dialysis effluent was collected. The secretion of TGF-β_1_ in the peritoneal dialysis effluent was determined by a TGF-β_1_ enzyme-linked immunosorbent assay (ELISA) kit according to the manufacturer’s instructions. The OD was determined by spectrophotometry.

### Statistical analysis

Values are expressed as the mean ± standard deviation. The data were assessed by one-way analysis of variance (ANOVA) followed by Tukey’s post-hoc test among different experimental groups. A statistically significant difference was defined at *p* < 0.05 using the statistical package SPSS for Windows 20.0 (SPSS, Chicago, IL).

## Results

### Reduced mitochondrial biogenesis capacity in a mouse model of PF

To determine the effect of PD-related PF on mitochondrial biogenesis and the effect of activation of the AMPK-PGC-1α pathway on PF, a model of PD-induced PF in mice was established, and adenovirus-mediated overexpression of PGC-1α was transfected into the peritoneum. Immunoblot, RT–PCR and immunohistochemistry were used to evaluate the expression of molecules involved in AMPK-PGC-1α signaling. As shown in Figure 1(A), the protein expression of phospho-AMPK (0.35 ± 0.20 *vs.* 0.67 ± 0.21), PGC-1α (0.61 ± 0.02 *vs.* 0.96 ± 0.23), NRF-1 (0.41 ± 0.04 *vs.* 0.88 ± 0.004), NRF-2 (0.45 ± 0.14 *vs.* 0.84 ± 0.14) and TFAM (0.51 ± 0.05 *vs.* 0.81 ± 0.10) was decreased in the PF group compared to the control group (*p* < 0.05). Activation of the AMPK-PGC-1α pathway (PGC-1α overexpression or metformin, which is an agonist of AMPK) increased the expression of phospho-AMPK, PGC-1α, NRF-1, NRF-2 and TFAM compared to that in the PF group (*p* < 0.05). In addition, RT–PCR analysis showed a decrease in PGC-1α mRNA in the PF group (shown in Figure 1(B)), and immunohistochemistry analysis showed a decrease in NRF-1 and NRF-2 in the PF group (shown in Figure 1(C,D)). PGC-1α overexpression or metformin treatment alleviated the altered expression of PGC-1α, NRF-1, NRF-2 and TFAM. Furthermore, RT–PCR was used to evaluate the mtDNA amount, which is an effective indicator of mitochondrial biogenesis. As shown in Figure 1(E,F), the ratio of COX I or COX II to 18S rDNA was decreased by 89% or 95% in the PF group compared to the control group (*p* < 0.05, respectively). PGC-1α overexpression or metformin treatment elevated the relative expression of COXI or COXII to 18S rDNA compared to that in the PF group (*p* < 0.05, respectively).

Figure 1.Reduced mitochondrial biogenesis capacity in a mouse model of PF. A mouse model of PD-induced PF was established. Animals were randomly divided into five groups: the control group, peritoneal fibrosis group (PF), peritoneal fibrosis + metformin group (PF + Met), peritoneal fibrosis + PGC-1α overexpression group (PF + PGC-1α), and peritoneal fibrosis + empty vector group (PF + Vector). (A) Immunoblotting was used to evaluate the expression of p-AMPK, PGC-1α, NRF-1, NRF-2 and TFAM. (B) RT–PCR was used to evaluate the mRNA expression of PGC-1α. (C&D) Immunohistochemistry was used to evaluate NRF-1 and NRF-2 expression (magnification ×400). (E&F) RT–PCR was used to evaluate the mtDNA amount. The ratio of COX I or COX II to 18S rDNA was calculated, indicating the relative mitochondrial copy number. The obtained results are representative of three independent experiments. *p < 0.05 *vs*. Control. ^#^*p* < 0.05 *vs.* PF. PD: peritoneal dialysis; p-AMPK: phospho-adenosine monophosphate-activated protein kinase; PGC-1α: peroxisome proliferator-activated receptor γ coactivator-1α; NRF: nuclear respiratory factor; TFAM: mitochondrial transcription factor A. RT–PCR: real-time polymerase chain reaction; mtDNA: mitochondrial DNA; COX: cytochrome c oxidase.

**Figure:**
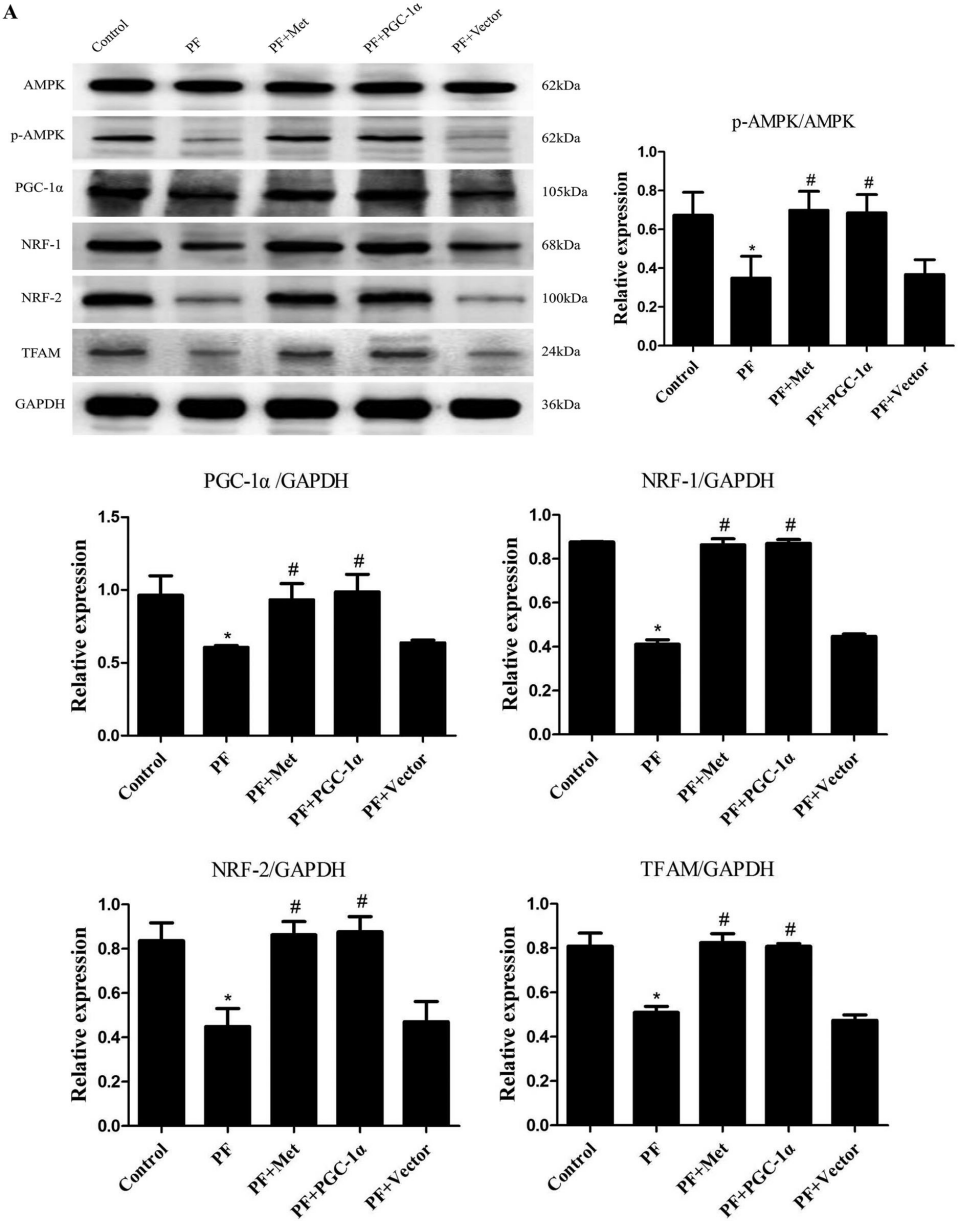

**Figure:**
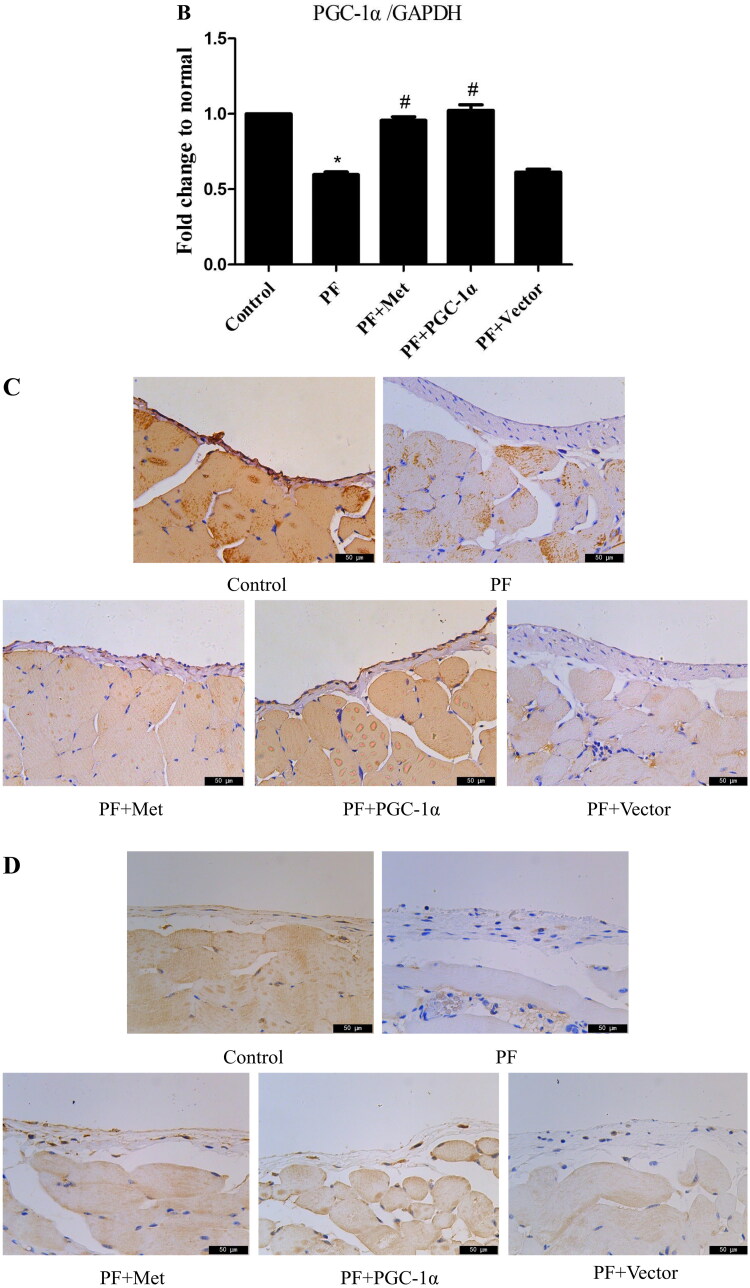

**Figure:**
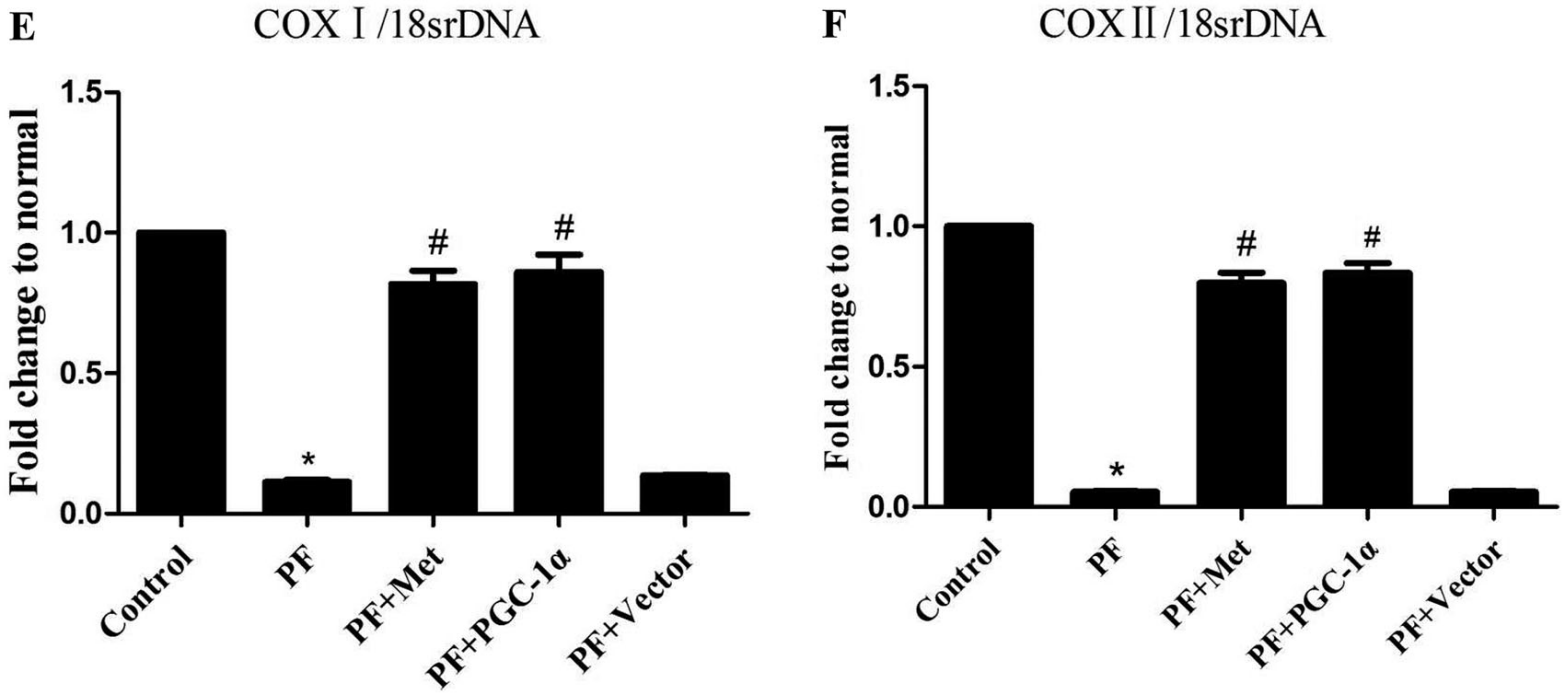

### Mitochondrial alterations in a mouse model of PF

**Figure 2.**
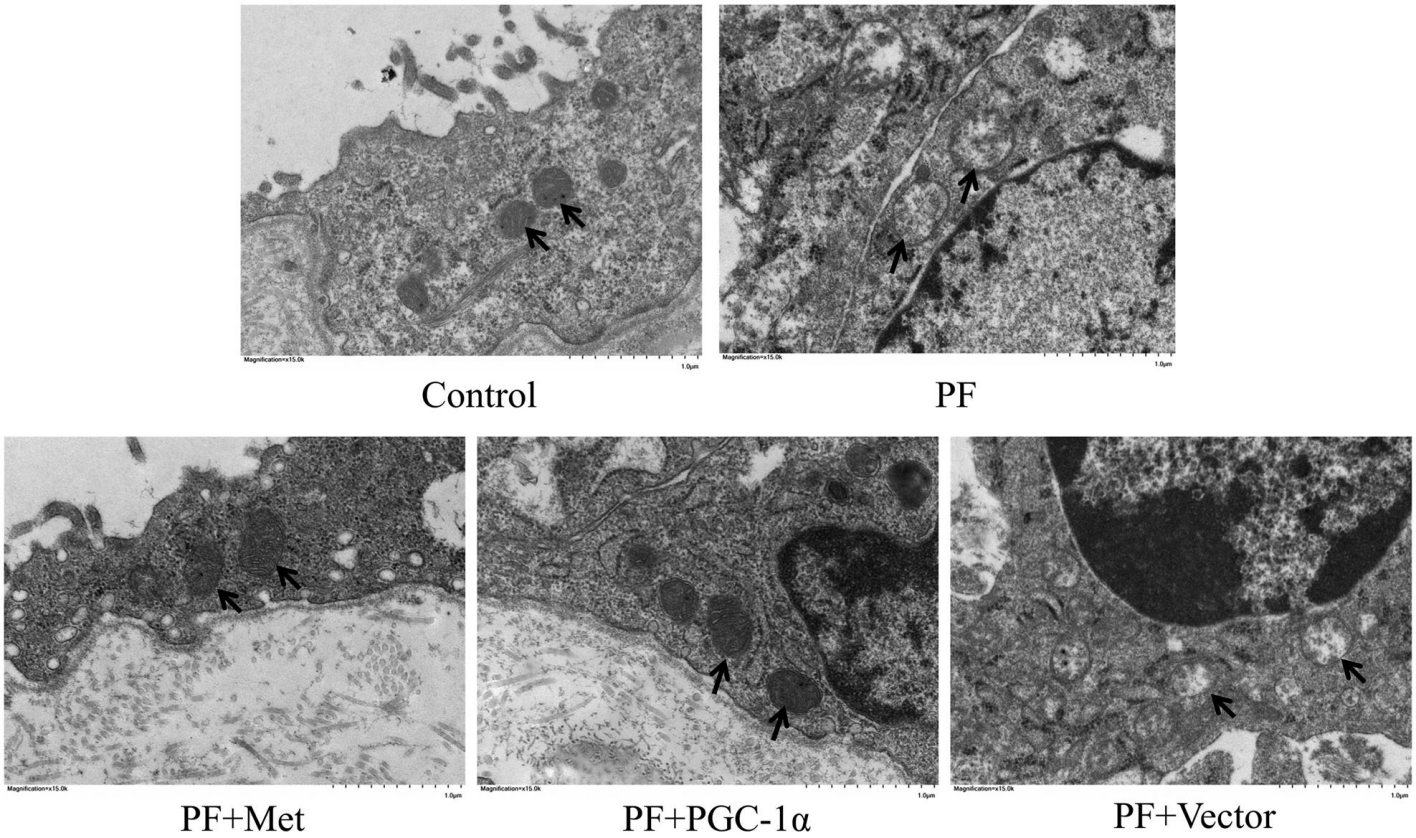
Mitochondrial alterations in a mouse model of PF. To determine the effect of PF on mitochondrial morphology, we observed the mitochondrial changes in PMCs in parietal peritoneal tissue using TEM. Typical TEM images are displayed (magnification ×15,000). PMCs: peritoneal mesothelial cells; TEM: transmission electron microscopy; PF: peritoneal fibrosis; PF + Met: peritoneal fibrosis + metformin; PF + PGC-1α: peritoneal fibrosis + PGC-1α overexpression; PF + Vector: peritoneal fibrosis + empty adenoviral vector.

To determine the effect of PF on mitochondrial morphology, we observed the mitochondrial changes in PMCs in parietal peritoneal tissue using TEM. As shown in Figure 2, PMCs showed a normal tightly packed mitochondrion appearance in the control group. In the PF group, mitochondria displayed a swollen appearance with collapsed cristae or even loss of discernable cristae. PGC-1α overexpression or metformin treatment alleviated these mitochondrial changes.

### Activation of the AMPK-PGC-1α pathway alleviates PF

Immunoblotting was used to evaluate the effect of activation of the AMPK-PGC-1α pathway on the expression of fibrosis molecules, including fibronectin, α-SMA and E-cadherin, in the visceral peritoneal membrane. As shown in Figure 3(A), the protein expression of fibronectin (0.51 ± 0.03 *vs.* 0.26 ± 0.06) and α-SMA (0.70 ± 0.09 *vs.* 0.48 ± 0.05) was increased in the PF group compared to the control group (*p* < 0.05). The protein level of E-cadherin (0.71 ± 0.06 *vs.* 1.03 ± 0.06) was decreased in the PF group compared to the control group (*p* < 0.05). PGC-1α overexpression or metformin treatment decreased the expression of fibronectin and α-SMA compared to the PF group (*p* < 0.05, respectively) and increased E-cadherin expression compared to the PF group (*p* < 0.05, respectively). In addition, Masson’s trichrome staining of the parietal peritoneum showed clear thickening of the mesothelium and submesothelial interstitium in the PF group compared to the control group (40.64 ± 7.73 μm *vs.* 13.78 ± 2.46 μm, *p* < 0.05, shown in Figure 3(B)). PGC-1α overexpression or metformin treatment resulted in a decrease in peritoneal thickness compared to that of the PF group (21.77 ± 4.29 μm *vs.* 40.64 ± 7.73 μm; 20.22 ± 3.92 μm *vs.* 40.64 ± 7.73 μm, *p* < 0.05, respectively, shown in Figure 3(B)). TGF-β_1_ is a key fibrogenic molecule of PF. We also investigated TGF-β_1_ expression in the peritoneal dialysis effluent using ELISA. As shown in Figure 3(C), the protein level of TGF-β_1_ was elevated in the PF group compared to the control group (395.85 ± 57.15 pg/mL *vs.* 160.67 ± 33.62 pg/mL, *p* < 0.05). PGC-1α overexpression or metformin treatment reduced TGF-β_1_ expression compared to that in the PF group (182.60 ± 41.15 pg/ml *vs.* 395.85 ± 57.15 pg/mL; 181.56 ± 51.57 pg/mL *vs.* 395.85 ± 57.15 pg/mL, *p* < 0.05, respectively).

Figure 3.Activation of the AMPK-PGC-1α pathway alleviates PF. (A) Immunoblotting was used to evaluate the effect of activation of the AMPK-PGC-1α pathway on the expression of fibrosis molecules, including fibronectin, α-SMA and E-cadherin, in the visceral peritoneal membrane. (B) Masson’s trichrome staining was used to measure the degree of PF. Representative histology of parietal peritoneal tissue is shown with a quantitation bar of peritoneal thickness (magnification ×400). (C) TGF-β_1_ expression in the peritoneal dialysis effluent was determined by ELISA. The results are representative of three independent experiments. *p < 0.05 *vs.* Control. ^#^*p* < 0.05 *vs.* PF. α-SMA: α-smooth muscle actin; ELISA: Enzyme-linked immunosorbent assay; PF: peritoneal fibrosis; PF + Met: peritoneal fibrosis + metformin; PF + PGC-1α: peritoneal fibrosis + PGC-1α overexpression; PF + Vector: peritoneal fibrosis + empty adenoviral vector.

**Figure:**
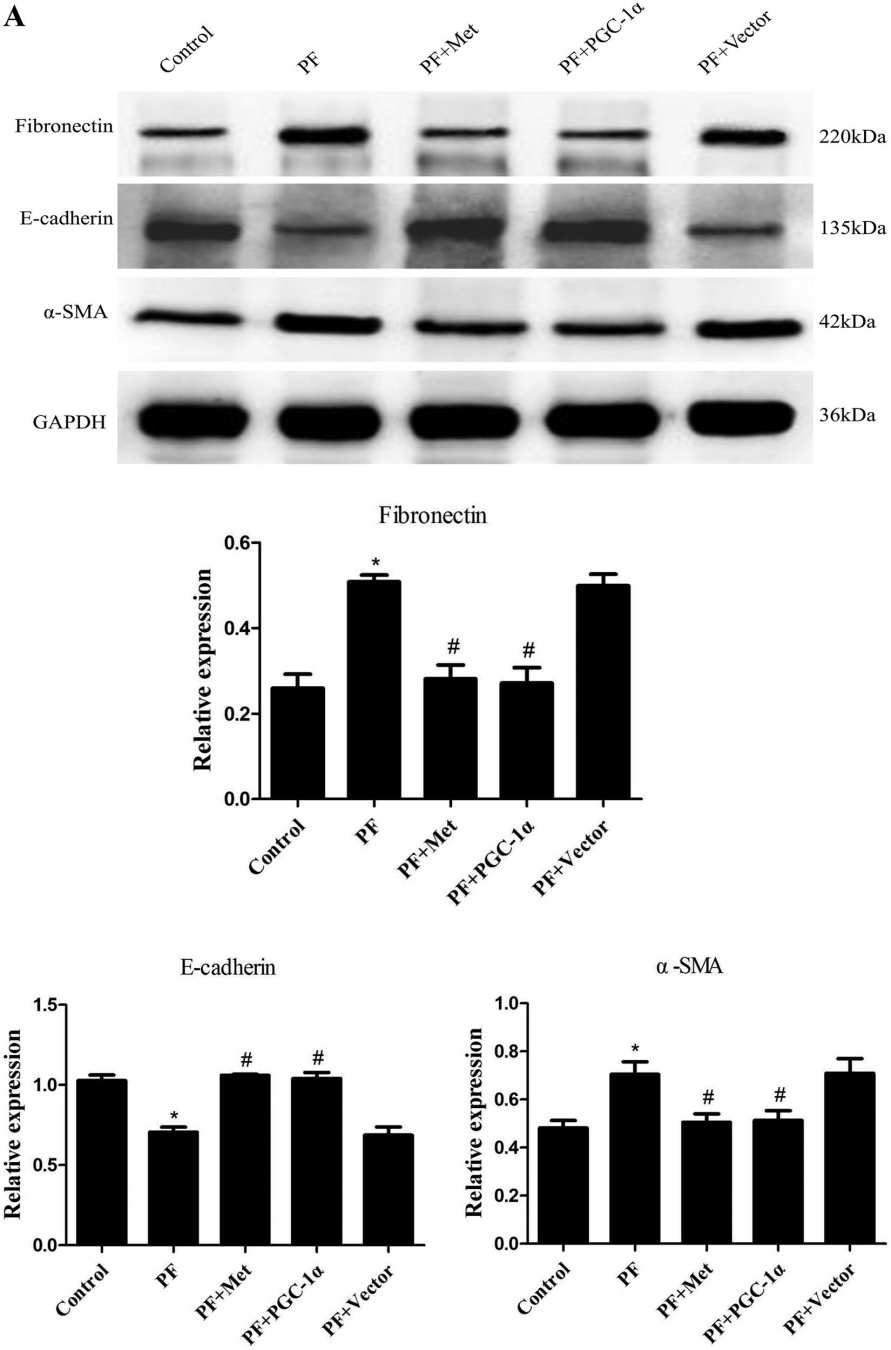

**Figure:**
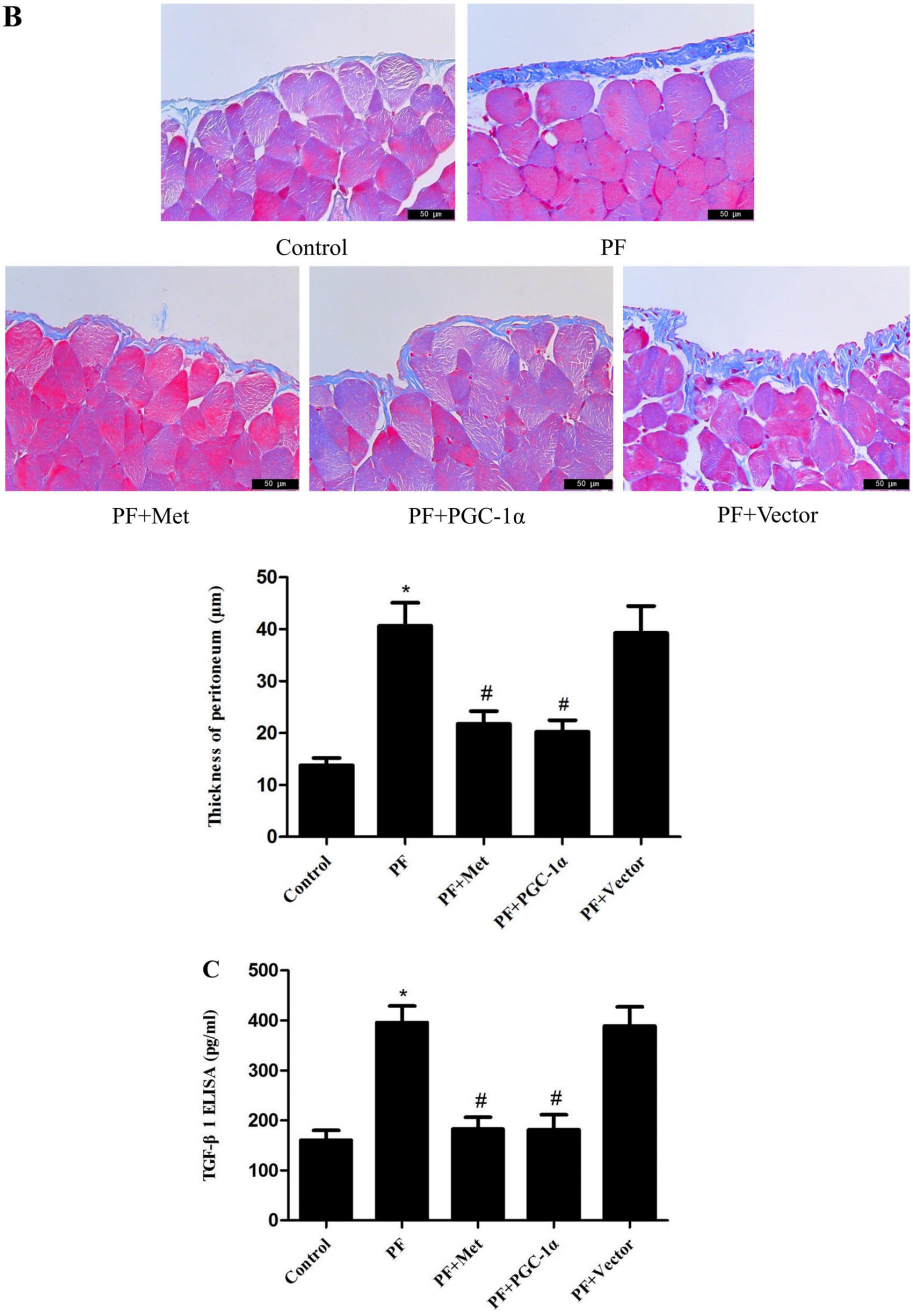

### Activation of the AMPK-PGC-1α pathway inhibited the apoptosis of peritoneal mesothelial cells

**Figure 4.**
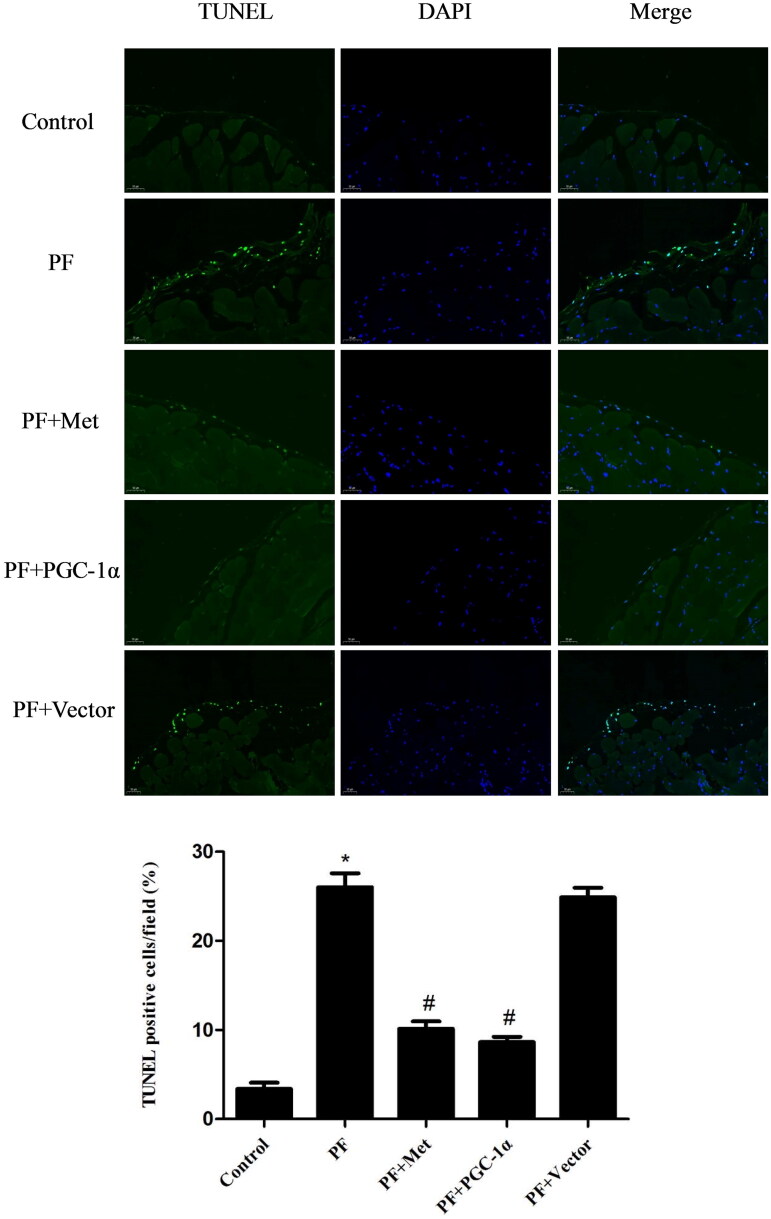
Activation of the AMPK-PGC-1α pathway inhibited the apoptosis of PMCs. The apoptosis of PMCs in parietal peritoneal tissue was revealed using a TUNEL assay. Representative images are displayed (magnification ×400). Quantitative analysis of TUNEL-positive cells. The results are representative of three independent experiments. *p < 0.05 *vs.* Control. ^#^*p* < 0.05 *vs.* PF. PMCs: peritoneal mesothelial cells; TUNEL: TdT-mediated dUTP nick-end labeling; PF: peritoneal fibrosis; PF + Met: peritoneal fibrosis + metformin; PF + PGC-1α: peritoneal fibrosis + PGC-1α overexpression; PF + Vector: peritoneal fibrosis + empty adenoviral vector.

Recent *in vitro* and *in vivo* studies demonstrated that apoptosis of PMCs caused by high glucose PD solutions played a role in the genesis of fibrosis [21]. We evaluated the apoptosis of PMCs in parietal peritoneal tissue using TUNEL assay. TUNEL staining of the parietal peritoneum showed typical apoptosis characteristics in the mesothelium in the PF group. PGC-1α overexpression or metformin treatment resulted in a reduced signal during fluorescence microscopyanalysis (shown in Figure 4).

## Discussion

The pathogenesis of PD-induced PF is not clearly understood, and current treatment options are limited. Recent studies have demonstrated that mitochondrial dysfunction participates in the development of organ fibrosis and considered mitochondria as a potential therapeutic strategy [13–16]. Very few articles have reported that restoring mitochondrial synthesis protein expression and mitochondrial function can inhibit peritoneal fibrosis [17,18]. Our previous study confirmed that high glucose-based PD fluids increased mitochondrial reactive oxygen species (ROS) production, which led to NLRP3 inflammasome activation and subsequent IL-1β secretion in human PMCs. Induction of mitophagy decreased mitochondrial ROS production, NLRP3 inflammasome activity and IL-1β expression. We concluded that induction of mitophagy may protect human PMCs from ROS-NLRP3-mediated peritoneal inflammatory injury, which plays an important role in the pathogenesis of PD-induced PF [22].

In this study, we observed the downregulation of PGC-1α signaling, which was manifested by decreased protein expression of PGC-1α, NRF-1, NRF-2 and TFAM and PGC-1α mRNA expression in high glucose-based PD fluid-induced peritoneal fibrosis. Furthermore, we found that the ratio of COX I and COX II to 18S rDNA was decreased, which indicated reduced mtDNA content. These data revealed that the capacity of mitochondrial biogenesis was impaired in the process of PD-related PF. Miao et al. [23] observed the downregulation of PGC-1α and TFAM and the decline in mtDNA content in a mouse model of age-related renal fibrosis. Krishnasamy et al. [24] reported that PGC-1α and TFAM expression were reduced, which indicated disruption of mitochondrial biogenesis in nonalcoholic steatohepatitis (NASH) and early fibrosis in mouse liver. Shao et al. [25] confirmed that the protein levels of PGC-1α, NRF-1 and TFAM were decreased in a mouse model of diabetes mellitus-related cardiac fibrosis. These results were in accordance with our current study. Together with previous reports, we presumed that mitochondrial biogenesis was inhibited in the process of organ fibrosis.

Mitochondria are unique double-membrane organelles that play essential roles in adenosine-triphosphate (ATP) production and a series of cellular signaling events. Preservation of mitochondrial morphology and ultrastructure is of vital importance. Herein, we observed mitochondrial changes in PMCs in parietal peritoneal tissue using TEM. PMCs showed a normal tightly packed mitochondrion appearance in the control group. In the PF group, mitochondria displayed a swollen appearance with collapsed cristae or even loss of discernable cristae. PGC-1α overexpression or metformin treatment alleviated these mitochondrial changes. This result indicated that morphological and mitochondrial impairment in PF and activation of the AMPK-PGC-1α pathway could potentially preserve peritoneal membrane mitochondrial morphology under PF conditions. Yu et al. [26] illustrated swollen and damaged mitochondria with severely disrupted cristae and membranes in a mouse model of lung fibrosis. Interestingly, the authors found that thyroid hormone treatment could reverse these mitochondrial changes in a manner dependent on PGC-1α. Zhang et al. [27] revealed malformed mitochondria appearing as balloon-shapes or small globules with few cristae in a mouse model of liver fibrosis. Liao et al. [28] observed mitochondrial matrix swelling and mitochondrial membrane disruption in unilateral ureteral obstruction (UUO) and renal ischaemia–reperfusion injury (IRI)-induced renal fibrosis. Furthermore, they found that fluorofenidone treatment could clearly improve mitochondrial morphological manifestations by improving mitochondrial biogenesis with upregulation of PGC-1α, NRF-1 and TFAM. We obtained similar results to these reports.

We explored the effect of activation of the AMPK-PGC-1α pathway on PF. We observed reduced protein levels of fibronectin and α-SMA and elevated protein levels of E-cadherin in the PGC-1α overexpression and metformin treatment groups compared with the PF group. We also demonstrated that PGC-1α overexpression or metformin treatment resulted in a clear decrease in peritoneal thickness and reduced TGF-β_1_ in peritoneal dialysis effluent compared to the PF group. All above mentioned results revealed that activation of the AMPK-PGC-1α pathway alleviated PF. Liao et al. [28] revealed that fluorofenidone treatment relieved renal fibrosis partly by improving mitochondrial biogenesis with increases in PGC-1α, NRF-1 and TFAM. Rangarajan et al. [29] confirmed that activation of AMPK by metformin reversed lung fibrosis in mice and that this effect was related to enhanced mitochondrial biogenesis. Jia et al. [30] reported that postinfarction exercise training alleviated myocardial fibrosis *via* mitochondrial biogenesis and PGC-1α signaling. Therefore, we can assume that activation of AMPK-PGC-1α signaling has a protective role in the development of organ fibrosis.

However, there is one limitation in this study. AMPK is a well-recognized metabolic regulator and cellular bioenergetic sensor [31,32]. Activation of AMPK by metformin has a broad range of pharmacological effects such as antioxidant, anti-inflammatory and mitochondrial membrane potential maintenance effects [33]. These effects are associated with the antifibrotic function. Therefore, we cannot conclude that the amelioration of PF is completely due to metformin-mediated mitochondrial biogenesis *via* PGC-1α signaling.

In conclusion, this study may provide the first evidence that mitochondrial biogenesis was impaired, and mitochondrial structure was damaged in the peritoneum in a mouse model of PD fluid-induced PF. Activation of the AMPK-PGC-1α pathway upregulated phospho-AMPK, PGC-1α, NRF-1, NRF-2 and TFAM expression and mtDNA content, improved mitochondrial morphological manifestations, inhibited apoptosis of PMCs and alleviated PF by enhancing mitochondrial biogenesis.

## Data Availability

The data that support the findings of current study are available from the corresponding author upon reasonable request.
